# Treating the mind to improve the heart: the summon to cardiac psychology

**DOI:** 10.3389/fpsyg.2015.01101

**Published:** 2015-08-04

**Authors:** J. P. Ginsberg, Giada Pietrabissa, Gian Mauro Manzoni, Gianluca Castelnuovo

**Affiliations:** ^1^Research and Development, Cardiopsychology Research Laboratory, Dorn VA Medical CenterColumbia, SC, USA; ^2^Psychology Research Laboratory, Istituto Auxologico Italiano IRCCSOspedale San Giuseppe, Verbania, Italy; ^3^Department of Psychology, Catholic University of MilanItaly

**Keywords:** HRV, heart rate variability, cardiac psychology, autonomic control of cardiovascular system, stress, psychological, PTSD

The link between the heart and mind has been studied over the centuries in many fields, such as medicine and psychology (Thayer and Lane, 2009; Allan, 2012). Different terms have been used recently to identify this research and clinical area: e.g., Behavior Cardiology (Rozanski et al., 2005), Psychocardiology (Jordan et al., 2007), and Cardiac Psychology (Allan, 2012). Many risk factors for coronary heart disease have been studied: thus, age, gender, and family history are considered as typical unmodifiable risk factors, whereas diabetes, weight, life stress, type A behavior, social isolation, depression, sedentary lifestyle, cholesterol/HDL ratio, hypertension, and cigarette smoking are the typical modifiable and clinically treatable risk factors (Allan, 2012).

Mental stress is now also recognized as a risk factor in cardiac dysregulation. Due to an “epigenetic psychobiologic susceptibility-the nexus of psychophysiologic reactivity and biopsychosocial vulnerability” (Fischer and Collins, 2012, p. 58), acute emotional traumas could “trigger a panic attack in some and transient or permanent cardiac damage or life-threatening arrhythmias or death in others” (Fischer and Collins, 2012, p. 58). Moreover mental stress could be an important trigger of cardiovascular events with clinical relevance, taking into account that stress responses can be mediated or moderated by psychological variables, such as coping skills or personality characteristics, and by social aspects such the presence of family or systemic support (Krantz et al., 2012). In many situations multiple stressors could be involved in generating cardiovascular events (Menezes et al., 2011). Natural calamities, such as earthquakes, and human-made extreme disasters, such as war and terrorism, can precipitate cardiovascular events (Mittleman and Mostofsky, 2012).

A typical field of intervention for the Cardiac Psychology is Posttraumatic Stress Disorder (PTSD), classified as an anxiety disorder in the latest version of Diagnostic and Statistical Manual of Mental Disorders (American Psychiatric Association, 2013). The essential feature of PTSD is the development of specific symptoms, following exposure to one or more traumatic events, such as recurrent and intrusive memories or dreams about the trauma (re-experiencing symptoms), flashbacks, emotional numbing, or heightened physiologic arousal. PTSD affects significant organic outcomes too (Doerfler and Paraskos, 2012). A typical example is reported in Shemesh et al. (2004), where a group of patients with PTSD followed one year after a myocardial infarction (MI) were more than twice as likely to be submitted to another hospitalization because of cardiovascular reasons than individuals with MI but without PTSD. Taking into account that many effective interventions are available nowadays to treat and reduce PTSD in different populations (Arnberg and Johannesson, 2013; Barrera et al., 2013; Gillies et al., 2013; Warner et al., 2013; Cukor and Difede, 2014), it appears to be the case that research into cardiac effects of PTSD is not highly developed, in fact in 2012 Doerfler wrote that “Research on CBT (Cognitive Behavioral Therapy) for cardiac-related PTSD is in its infancy, and the literature consists of only a few uncontrolled case studies, but these reports may be instructive in stimulating treatment development” (Doerfler and Paraskos, 2012, p. 260).

So the study of psychosocial factors and interventions in the field of Cardiac Psychology and PTSD is a growing need and challenge in our clinical and scientific community (Ginsberg et al., 2008, 2010; Chen et al., 2014; Conder and Conder, 2014; Drury, 2014; Gillie and Thayer, 2014; Lee et al., 2014; Lehrer and Gevirtz, 2014; Mccraty and Zayas, 2014; Shaffer et al., 2014; Steffen et al., 2014; Wood, 2014). In cardiovascular rehabilitation protocols it is important to evaluate different clinical psychology-based program types, such as psychological interventions, psycho-educational programs, psychotherapies, educational training, stress management, biofeedback, counseling sessions and relaxation techniques (Jordan et al., 2007; Dornelas, 2008, 2012; Manzoni et al., 2008; Castelnuovo, 2010a,b). New approaches have to be tested, such as Acceptance and Commitment Therapy (Spatola et al., 2014a,b) or expressive writing (Manzoni et al., 2011a), improving the study of rehabilitation programs on patients with comorbidities such as obesity (Manzoni et al., 2011b; Pietrabissa et al., 2012), improving the study of psychosocial and cognitive features related to the cardiac pathology with or without complications (Capodaglio et al., 2010, 2013; Manzoni et al., 2010, 2011a; Proietti et al., 2012, 2014; Cazard and Ferreri, 2013; Castelnuovo et al., 2014), and opening to the growing opportunities provided by new technologies and mHealth approach (Castelnuovo et al., 2003, 2014; Nguyen et al., 2004; Rubel et al., 2005; Riva et al., 2006; Roth et al., 2009; Castelnuovo and Simpson, 2011).

The study of the relationship of autonomic cardiac adjustment to stress and mental disorder—the heart-mind connection—is the challenge that Cardiac Psychology has accepted. This *Frontiers* Research Topic special is devoted to providing a foundation for the development of the scientific study of these relationships, and the discovery of the propositions that govern them.

## Conflict of interest statement

The authors declare that the research was conducted in the absence of any commercial or financial relationships that could be construed as a potential conflict of interest.
